# Ten Simple Rules for Aspiring Scientists in a Low-Income Country

**DOI:** 10.1371/journal.pcbi.1000024

**Published:** 2008-05-30

**Authors:** Edgardo Moreno, José-María Gutiérrez

**Affiliations:** 1 Programa de Investigación en Enfermedades Tropicales, Escuela de Medicina Veterinaria, Universidad Nacional, Heredia, Costa Rica; 2 Instituto Clodomiro Picado, Facultad de Microbiología, Universidad de Costa Rica, San José, Costa Rica

Being a scientist entails a common set of characteristics. Admiring nature and having concern for social issues; possessing a strong academic background, team work abilities, honesty, discipline, skepticism, communication skills, competitiveness, ability to accept and give criticism, and productive relationships are some of the most obvious traits that scientists should have. To be a scientist in a low-income country (LIC), however, requires a complementary set of qualities that are necessary to confront the drawbacks that work against the development of science. The failure of many young researchers to mature as professional scientists upon their return to their country from advanced training elsewhere, motivated us to propose these ten rules.

## Rule 1: Understand Your Country

Most LIC scientists want to live in their home country. Nevertheless, you must be realistic and prepared to face rudimentary laboratories, power cuts, poor water supply, deficient libraries, slow Internet, and scarce or non-existent national funds for supporting research, hiring personnel, and providing maintenance or equipment. You must understand that science is a minor component of the cultural environment of an LIC and that, for most people and many politicians, science is a curiosity performed in high-income countries [1]. Within this adverse scenario, you should establish broad and strong links with your community and country. This involves becoming interested in historical, social, and political issues. LIC researchers have to enjoy the idiosyncrasies of their country, and cultivate the desire to contribute to the scientific development of their homeland and to the well-being of its people. Do not endorse deep doubts about the possibilities of performing research. It can be done—but not alone. Try to join efforts with other investigators facing the same problems. Learn how they sidetrack difficulties, and incorporate yourself into a research team. If you are not able to find a group that fits your specific interest, then procure a group of researchers who, although investigating topics marginal to your own, are capable of understanding the relevance of your work. At the initial phases of your career, belonging to a creative scientific environment in which your knowledge and skills are appreciated is of major importance. Be part of a team before trying to lead one.

## Rule 2: Focus on Your Scientific Work

Your formal education has finished, but your scientific career is just beginning. Research should be your main professional activity. Consider that you may be the country's only specialist in a particular topic, but keep in mind that science is global. You are a small fish in a big pond and part of an international community. Grow within this global context. Concentrate on your work, and do not pay attention to flattering comments. Above all, keep away from activities that distract you from scientific endeavor, such as excessive administrative duties, and too many committees. Limit the number of meetings and attend only the relevant ones. Even though you are well prepared, modestly declare yourself as “ignorant” in topics that may distract you, and fight against excessive lecturing. However, participate in graduate programs and seminars. This is the right environment for the promotion of academic knowledge and skills.

## Rule 3: Be Wise When Selecting Your Research Topic

LICs face many problems that await creative solutions. Bizarre as it sounds, you can turn this into an advantage since these same problems constitute excellent sources for research and offer comparative advantages. Try to choose a topic that is not directly pursued by many or strong international research teams. At the beginning of your career, you cannot compete with them and your efforts may be frustrated. Identify the potential bottlenecks. Remember that in LICs research time runs slower and that good science is not so much related to the subject as to the answers you extract from your investigations. Frequently, local models become universal once a coherent story is built around them. Become an expert and, simultaneously, broaden your knowledge in collateral areas that may open new possibilities.

## Rule 4: Improve Your Communication Skills

English is the language of natural sciences, and you cannot avoid this fact. Consequently, you should be proficient in this language. The international scientific community is lenient about strong accents. However, the same community does not tolerate poor writing. Thus, writing skills are essential, since research begins with written proposals [2] and does not end until your results have been published [3]. You, more than native English speakers, must practice your oral presentations [4].

## Rule 5: Collaborate Locally and Internationally

Collaboration is essential for the advancement of science. Although this holds true for any researcher in the world [5], it is crucial for LIC investigators. Identify local groups who share your scientific interest, have equipment, or perform activities or techniques that are useful for your research. Keep in touch with your former tutor and colleagues and explore new collaborations abroad. Do not be shy about requesting help, and offer something that attracts the attention of your counterparts. Attend international meetings and present your work. Research is, in a way, a trade market of ideas, methods, and goods. Travel and visit research institutions. If some experiments cannot be carried out in your country, arrange to perform them abroad, or convince people to do them for you. There are international funds available for this purpose.

## Rule 6: Commit Yourself to the Education of Young Scientists

LIC researchers should participate in graduate training programs since this is the best way to build a strong scientific community. It is also a way to identify good young students and potential partners. Carefully choose the subjects for your students, pondering the possibilities of your research center, and be realistic about what they can achieve and the tasks you are imposing on them. Upgrade your students' education by sending them abroad for seminars and for learning specific methodologies (http://iscbsc.org/scs3/index.htm). There are international fellowships for this purpose (http://www.twas.org/). Be strict but generous with your students and colleagues, and, whenever possible, share your facilities and knowledge. Do not be self-centered. Promoting the success of others is also a way to promote your own success.

## Rule 7: Write Research Grants and Publish in International Journals

Scientific amateurism is common in LICs. Science is not a hobby but a professional activity that requires strong commitment. Inform yourself about local and international granting agencies, and apply for money [2]. There are international agencies and programs that provide grant and travel funds for LIC investigators (e.g., TWAS, IFS, EU, NIH, etc.). Although funds are limited, they will help you to build your scientific career. Incorporate yourself into international consortia; they may find your ideas and resources interesting. If you do not have access to essential publications, send requests to authors, editors, or colleagues abroad. Avoid publishing your results in magazines or low-quality journals, and instead submit your work to international journals. Do not overestimate or underestimate your work, be realistic when choosing a suitable journal [3], and, above all, do not be overly frustrated when grants or papers are rejected; instead, use the experience as a source of learning. Even though some reviewers may undervalue research performed in LICs, most of them pay more attention to the results and ideas than to nationalities [6].

## Rule 8: Develop Endurance When Confronting Difficulties

It is understandable that the limitations of performing research in LICs sometimes weaken your enthusiasm. Remain calm and try to identify the source of the problem; avoid complaining excessively in front of students, colleagues, or your partners abroad. A negative attitude is contagious, lowers your prestige, and has the tendency to attract unproductive people. Share your problems with other local scientists and confront them as a team. You should cultivate your abilities to find alternative solutions, as well as skills to improvise and to persuade people.

## Rule 9: Educate Yourself as a Professional Scientist

To be a specialist in an LIC is not enough. Be aware that the scientific community in an LIC is in short supply and lacks redundancy. In order to confront the drawbacks and deficiencies of the system, you must acquire a wide scientific knowledge, and become a well educated person in a broad sense. In addition to helping the quality of your research, this will give you the credentials to participate in political decisions related to science, to promote your ideas, and to spread scientific knowledge in your country. Acquaint yourself with local and international trends related to scientific performance and keep track of the major breakthroughs in science. Give talks and write about science whenever you consider it pertinent, but without diverting your attention too much from your main scientific duties.

## Rule 10: Appreciate Being a Scientist

As most scientists from high income countries and from LICs know, we are prone to facing economic difficulties at the beginning of our careers. Generally, salaries for scientists are comparatively low. Nevertheless, in time scientists can achieve a satisfying income; furthermore, there are compensations, especially if you become a successful scientist. A sense of achievement and contribution to your community, prestige, travel, meeting interesting people, and consulting opportunities are some of them, but nothing is more rewarding than the intellectual stimulation of science itself. This was your original motivation; nourish it with more and better science.

## References

[1] Moreno E, Alveteg T (2002). Collaboration between Sweden and the Public Universities of Nicaragua.. http://www.oecd.org/dataoecd/43/21/35213123.pdf.

[2] Bourne PE, Chalupa LM (2006). Ten simple rules for getting grants.. PLoS Comput Biol.

[3] Bourne PE (2005). Ten simple rules for getting published.. PLoS Comput Biol.

[4] Bourne PE (2007). Ten simple rules for making good oral presentations.. PLoS Comput Biol.

[5] Vicens Q, Bourne PE (2007). Ten simple rules for a successful collaboration.. PLoS Comput Biol.

[6] Yousefi-Nooraie R, Shakiba B, Mortaz-Hejri S (2006). Country development and manuscript selection bias: a review of published studies.. BMC Med Res Methodol.

